# Metabolomic and physiological insights to ameliorate post-harvest stress in cultured mussels

**DOI:** 10.1007/s11306-025-02289-1

**Published:** 2025-06-26

**Authors:** M. C. F. Cheng, M. R. V. Welford, L. N. Zamora, N. J. Delorme, N. L. C. Ragg, A. J. R. Hickey, B. J. Dunphy

**Affiliations:** 1 School of Biological Sciences, University of Auckland, Private Bag 92019, Auckland, 1142 New Zealand; 2 Cawthron Institute, Private Bag 2, Nelson, 7042 New Zealand

**Keywords:** Metabolomics, Post-harvest stress, Emersion, *Perna canaliculus*, Anaerobic metabolism, Live transport

## Abstract

**Introduction:**

Survival and quality of Green-lipped mussels (*Perna canaliculus*) exported live could be further improved through enhanced post-harvest handling, aiming to reduce the physiological stress associated with transport out of water. Addressing these issues requires identifying treatments to reduce post-harvest stress and understanding underpinning molecular mechanisms.

**Objective:**

This study aimed to evaluate treatments (low temperature and MgCl_2_ anaesthetic baths) to mitigate post-harvest handling stress in mussels.

**Methods:**

We analysed metabolomic profiles using gas chromatography-mass spectrometry (GC/MS), anaerobic enzyme activity in gill and adductor muscle, and haemolymph biochemistry (pH, antioxidant capacity and osmolality) in mussels subjected to 14 °C, 4 °C or MgCl_2_ water-bath treatments after simulated harvest.

**Results:**

Metabolomic analyses revealed post-harvest mussels experienced increased anaerobic activity, osmotic and oxidative stress, reduced pH (Δ0.31), and lower polyunsaturated fatty acids (PUFA). Mussels immersed in 14 °C seawater recovered from anaerobiosis but had a strong indication of oxidative stress. Although mussels in 4 °C immersion had increased levels of PUFA, implying depressed lipid oxidation, the treatment did not improve recovery from anaerobiosis, indicated by reduced pH (Δ0.38). Mussels treated with MgCl_2_ showed some recovery from anaerobic handling stress, with decreased anaerobic end product accumulation and a more modest haemolymph pH decline (Δ0.16) compared to controls. While anaerobic enzyme activities showed tissue-specific responses, they did not exhibit the pronounced differences among treatments shown by their products in metabolic profiling.

**Conclusion:**

Among the proposed re-immersion treatments, immersing mussels in seawater containing 40 g L^− 1^ MgCl_2_ seemed to be the most effective treatment to alleviate post-harvest metabolic stress, therefore potentially increasing shelf-life of mussels destined for live export.

**Supplementary Information:**

The online version contains supplementary material available at 10.1007/s11306-025-02289-1.

## Introduction

Global consumption of aquatic food products has increased almost six-fold to 158 million tonnes p.a. in the last 60 years (FAO, 2022). To meet growing demands, aquaculture has developed and improved to increase sustainability and reduce harvesting of wild populations and impacts on surrounding ecosystems (Stoner, 2013). Shellfish, and in particular low-trophic level filter-feeding bivalves, appear to be among the most sustainable aquaculture organisms, with high yield and low environmental footprints (Shumway et al., 2003; Yaghubi et al., 2021). 

Bivalves contribute to a major marine shellfish aquaculture economy, with global exports exceeding four billion USD (FAO, 2022). In New Zealand, the green-lipped mussel, *Perna canaliculus*, is the most important shellfish species farmed, representing 96% of the local total bivalve production (King & Lake, 2013). In the past year, 30,000 tonnes of *P. canaliculus* were exported overseas, with a value of $380 million NZD (~$230 million USD; Aquaculture New Zealand, 2023) and the demand for green-lipped mussels is increasing.

Like most bivalve species, *P. canaliculus* experiences significant post-harvest stress involving aerial exposure (Nguyen et al., 2020). The health status and quality of live seafood traded are of major concern, which can be affected by post-harvest conditions (e.g., Day et al., 2022; Xu et al., 2021). For example, during the early post-harvest processes, tonnes of live mussels are removed mechanically from longlines, then washed, declumped, bagged in bulk containers and transported to processing factories (Hickman, 1991). Under such an environment, shellfish, including mussels, likely suffer from desiccation (osmotic dysregulation), hypoxia, thermal and oxidative stress, and an accumulation of metabolic wastes due to prolonged aerial exposure. All these insults result in decreased health status, if not shellfish mortality (Clements et al., 2018; Nguyen et al., 2020; Zamora et al., 2019), which may reduce live shellfish quality and undermine profitability of the aquaculture practice.

In the face of physiological challenges, i.e., unfavourable conditions, organisms may respond via metabolic re-organisation to enhance specific metabolic pathways to cope with changing demands (Tomanek, 2014; Wagner et al., 2015). Since metabolites are intermediates and end-products of metabolic pathways, changes in metabolic pathway activity levels can be revealed by the variations in the composition of metabolites of an organism. Previous studies showed that changes in the metabolic profiles of bivalves have been linked to perturbation by the external environment, such as elevated temperature (Dunphy et al., 2015, 2018; Jiang et al., 2020), hypoxia (Haider et al., 2020; Xu et al., 2021; Yang et al., 2023) and osmotic stress (Carregosa et al., 2014; Zhou et al., 2022). For example, the clam, *Corbula japonica*, upregulated metabolic pathways liberating free amino acids involved in osmoregulation in response to a hypersaline challenge (Koyama et al., 2015).

In a resource-limited (e.g., low dissolved oxygen and food) environment, organisms frequently shift metabolic pathways for energy transfer from aerobic to anaerobic (Anestis et al., 2010). While bivalves convert glucose to lactate, they can harness a similar group of enzymes, i.e., opine dehydrogenases, to maintain cellular redox state (NADH/NAD^+^) and ATP production during anaerobiosis (Livingstone, 1991). In molluscs, there are four key anaerobic pathways (i.e., glucose-succinate, aspartate-succinate, glucose-opine and glucose-lactate pathways; Livingstone, 1991). The end products of these anaerobic pathways, such as succinate, alanine, alanopine, octopine and strombine, can be used as indicators of the intensity of anaerobic metabolism in molluscs (Gäde, 1983a). For example, levels of opine end products strombine and octopine in the mussel, *Mytilus edulis*, increased three-fold after 24-hour aerial exposure (De Zwaan et al., 1983). Opine end products are produced through different opine dehydrogenases (Harcet et al., 2013), and changes in metabolite composition could also be attributed to variations in anaerobic enzyme activities. By complementing metabolic profile analyses with enzyme activities and activation, one can better elucidate the effects of perturbating the external environment on mussels (e.g., Jiang et al., 2023).

In addition to metabolomics and anaerobic enzymes, the body condition and health status of shellfish in response to stress can also be assessed by various biochemical parameters, such as pH and osmolality of haemolymph (Day et al., 2022; Hooper et al., 2014; Pérez–Velasco et al., 2022). Changes in such biochemical properties of haemolymph could be complementary to changes in metabolic profiles, providing a more holistic understanding of how shellfish respond to environmental stress. For instance, after 24 h of aerial exposure, the haemolymph pH of the crab *Lithodes santolla* dropped by 0.75, which is likely caused by an accumulation of carbonic acid and hydrolysis of ATP when oxygen is limited or absent (Lorenzo et al., 2020; Silverstein, 2021). The haemolymph osmolality of the clam, *Meretrix lusoria*, also decreased in hyposaline conditions, and this is likely associated with the decreased level of taurine, an important osmolyte for osmoregulation (Lin et al., 2021). Environmental stress could also elevate reactive oxygen species (ROS) production from multiple processes (Storey, 1996). Excess ROS can damage cellular components and negatively impact health, as an imbalance between ROS production and depletion through antioxidative defences results in oxidative stress (Lesser, 2006). Measuring total antioxidant capacity (TAC; i.e., the overall potential of an individual to combat ROS) can provide a measure of the health of an organism (Pisoschi et al., 2015) and measurement of haemolymph TAC has been used to indicate oxidative stress loads in the green lipped mussel, *Perna canaliculus* (Delorme et al., 2021a, b).

Effects of environmental stress associated with post-harvest handling conditions on *P. canaliculus* have been studied at whole organism and subcellular levels (Nguyen et al., 2020; Powell et al., 2017; Zamora et al., 2019). However, there have been few studies on how the negative effects associated with post-harvest handling can be mitigated (Anacleto et al., 2013; Barrento et al., 2013; Barrento & Powell, 2016; Zamora et al., 2019). Our previous work has shown that low temperatures and the anaesthetic and relaxant effects of magnesium chloride (MgCl_2_) decrease *P. canaliculus* metabolism (Cheng et al., 2024). Such treatments may therefore also mitigate the negative effects of post-harvest handling.

This study aimed to investigate the metabolic effects associated with post-harvest handling on the New Zealand green-lipped mussel, *P. canaliculus*, and to explore whether manipulation of these conditions can be used for post-harvest stress management. Here, we monitor the metabolome, activities of anaerobic enzymes, and haematological biochemistry with and without exposure to MgCl_2_, low temperature (4 ^o^C) and 14 ^o^C (i.e., the ambient condition to represent mussels without going through any stress mitigation conditions).

## Materials and methods

### Mussel collection and maintenance

Adult mussels (shell length ~ 65–100 mm; *n* = 100) were obtained from farms in the Marlborough Sounds (South Island, New Zealand) in July 2023. The mussels were subsequently transported to the Cawthron Aquaculture Park (Nelson, New Zealand) within 24 h, cleared of any biofouling organisms, and treated with freshwater in accordance with biosecurity guidelines (i.e., two-minute dip in freshwater, then hypochlorite solution, then re-immersion in freshwater). Mussels unable to close their valves during cleaning in freshwater, or displaying cracked shells, were discarded. The mussels were then allocated to three holding tanks (65 L) at 13.4 ± 0.8 °C (mean ± SD) in flow-through filtered seawater (1 μm-filtered; 36‰ salinity) with algae supply (1:1 *T-Isochrysis lutea* and *Chaetoceros muelleri*; 4.5 ± 1.1 µg Chl *a* L^− 1^) for two weeks to allow recovery from collection and transport prior to experimentation. The complete experiment was repeated three times. In each trial, mussels first went through simulated post-harvest (see Sect. 2.2) and were then exposed to different treatments (see Sect. 2.3).

### Simulation of post-harvest conditions

**Fig. 1:**
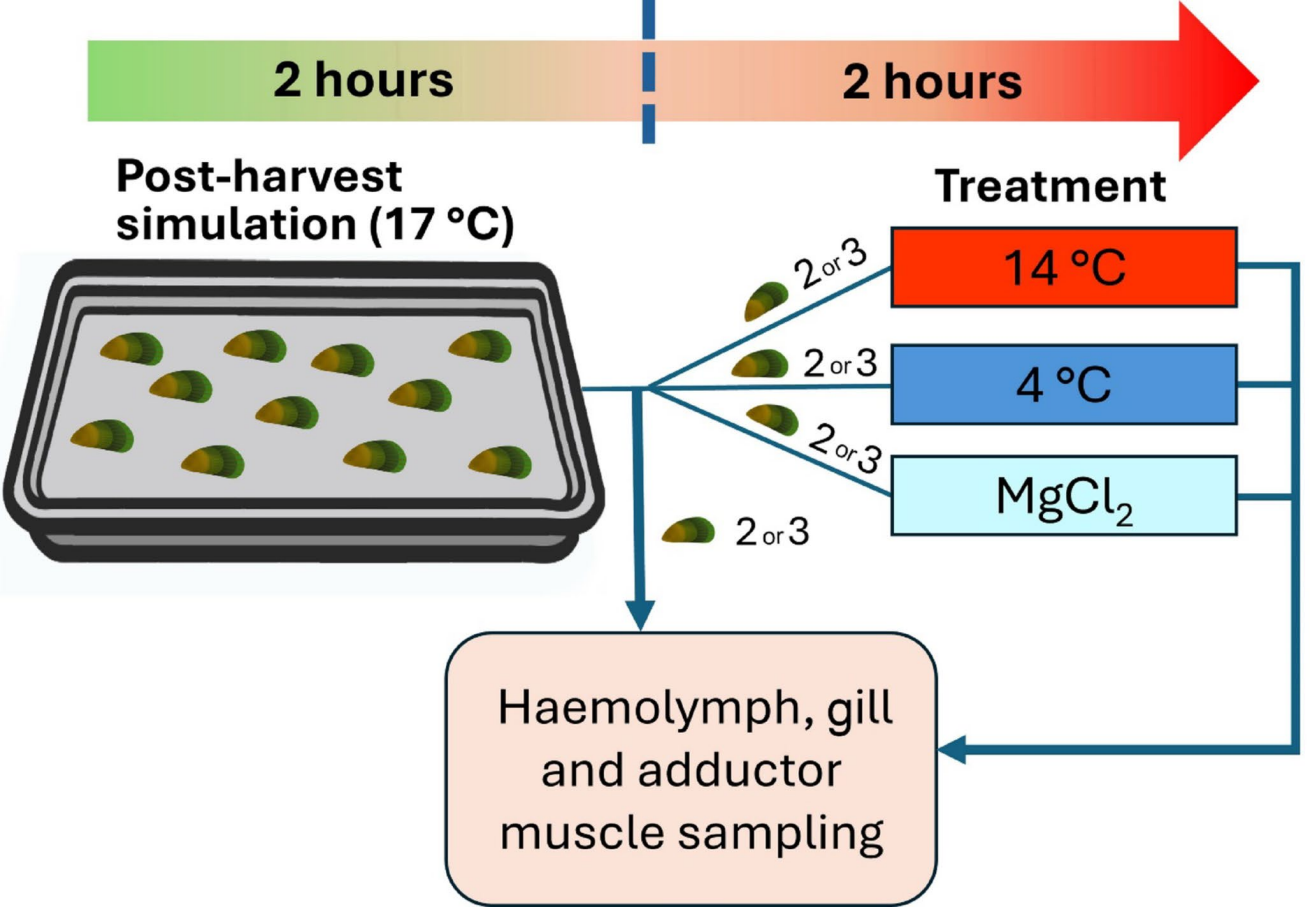
Diagram showing the experimental design of the present study for each trial. Mussels (*n* = 10–11) underwent post-harvest simulation (i.e., aerial exposure) for two hours, two to three mussels were sampled for haemolymph, gills and adductor muscle tissue. After post-harvest simulation, the mussels were assigned to and placed in different treatments (i.e., 14 °C, 4 °C and MgCl_2_ immersion) for two hours followed by haemolymph, gill and adductor muscle sampling. After three replicate trials, a total of eight mussels from each condition had been sampled

The experiment was conducted in three trials. In each trial, 10–11 mussels were randomly selected from the holding tanks and subjected to simulated post-harvest conditions. Mussels were placed in a shallow plastic tray (41 × 30 cm) positioned in a temperature-controlled room (~ 17 °C) for two hours, simulating the transport conditions from farm to factory (Fig. 1). All mussel individuals were positioned with their right valves facing downward (i.e., in contact with the tray) and posterior end facing to the right, to reduce any variation due to shell orientation. Following the two-hour aerial exposure, two to three mussels were sampled for haemolymph, gill and adductor muscle tissues (see Sect. 2.4). At the same time, two to three mussels from the holding tanks (control group) were also sampled. This process resulted in a total of eight mussels per treatment group (control and post-harvest) across three trials, resulting in 16 individuals overall (∑*n* = 2 groups × 8 mussels = 16 individuals). The remaining mussels were subsequently exposed to different treatments as described in Sect. 2.3 for an additional two hours. After treatments, haemolymph and tissue samples of these mussels were collected for analysis (Fig. 1), yielding eight mussels per treatment in total after three replicate trials. This structured approach ensured consistency and minimised variations in exposure durations across individuals (see Table 1 for the number of mussels used in each treatment per trial).

**Table 1:** The allocation of mussels for different conditions in each trial

Trial	Post-H	14 °C	4 °C	MgCl_2_	Total
1 (3)	3	3	2	3	11
2 (3)	3	3	3	2	11
3 (2)	2	2	3	3	10

The numbers in parentheses represent the number of mussels from the control group for each trial. The total number of mussels is the number of mussels used excluding the control

### Exposure to different re-immersion treatments for post-harvest stress mitigation

After the two-hour post-harvest simulation, mussels were divided into three treatment groups (two to three mussels per group per trial) to determine which treatment most effectively mitigated post-harvest stress. The treatments included immersion in chilled seawater at either 14 °C (14.3 ± 0.4 °C) or 4 °C (4.6 ± 0.1 °C), or a 40 g MgCl_2_ L^− 1^ solution (36‰ salinity) at 14 °C (14.6 ± 0.4 °C) for two hours with aeration (i.e., ∑*n* = 3 treatments × 8 mussels = 24 individuals for three trials), all of which suppressed *Perna canaliculus* metabolism within this period (Cheng et al., 2024). Temperatures were maintained using water baths connected to chillers (Hailea HC-500 A, China) and circulation pumps, with temperature monitored every minute by a thermocouple (Lutron, TM947SD, Taiwan). The MgCl_2_ solution was prepared by dissolving MgCl_2_·6H_2_O in a mixture of seawater (36‰) and freshwater (3:7 v/v) to account for the salinity with the added MgCl_2_. To assess the effect of treatment volume, mussels were also exposed to treatments in either 1 L–0.5 L volumes. However, no significant difference in metabolic responses was observed between the volumes (Fig. S1, S2), so 0.5 L was used for all subsequent treatments.

### Haemolymph extraction and tissue sampling

After simulated post-harvest and after exposure to the different immersion treatments, mussels were weighed and shell length was measured before sampling haemolymph, gill and adductor muscle. All samplings for each mussel took place within a five minute period to minimise the time effects on the biochemical signals in the samples. For sampling, mussel valves were slightly spread apart with a blunt knife and ~ 500 µL of haemolymph were extracted using a pre-chilled 1 mL syringe with a needle (25G × 38 mm) from the posterior adductor muscle (Fyhn & Costlow, 1975). Haemolymph was then transferred into 1.5 mL microcentrifuge tubes and stored in a cool box on ice while different parameters were measured (see Sect. 2.5.1 for details). Gills from both valves were sampled and separated into two samples for metabolomic profiling and anaerobic enzyme analysis. Each sample contained gills from both left and right valves. The posterior adductor muscle was bisected for the same analyses as the gills. All tissue samples were transferred into labelled 2 mL cryogenic tubes, snap-frozen in liquid nitrogen and stored in a freezer at -80 °C until analysis.

### Metabolic profiling

#### Metabolite extraction and derivatisation

Metabolic profiling was performed in the Mass Spectrometry Centre at the University of Auckland (UoA), New Zealand. Metabolic profiles of mussels were analysed using gas chromatography-mass spectrometry (GC-MS). Frozen tissues were dried overnight using a freeze-drier (Alpha 2–4 LD, Martin Christ, Germany). Then, the dried samples were homogenised at room temperature with a ceramic mortar and pestle. Approximately 10 mg of each sample was then transferred to a 1.5 mL microcentrifuge tube and mixed with 20 µL 10 mM d_4_-Alanine (L-alanine-2,3,3,3-d_4_) as an internal standard. All samples were extracted using cold methanol-water solutions, as previously described (Smart et al., 2010). Firstly, 500 µL of 50% cold methanol-water solution was added to each sample, followed by vigorous mixing in MiniG™ (1600, SPEX™ SamplePrep, USA) for one minute, followed by five-minute centrifugation (5424R, Eppendorf, Germany) at -9 °C, 3500 rpm (i.e., 1150 rcf). The supernatant was then transferred into 2 mL microcentrifuge tubes on dried ice. The above extraction processes were repeated one more time with 500 µL of 80% cold methanol-water solution and the supernatant was transferred to the same 2 mL microcentrifuge tubes. The 2 mL microcentrifuge tubes with the methanol-water solution containing metabolites were dried in a Speedvac concentrator (Savant™ SC250EXP, Thermo Scientific) connected to a refrigerated vapor trap (Savant™ RVT5105, Thermo Scientific) for four hours at 0.8 Hpa without heating. After drying, the samples were kept in a freezer at -80 °C before derivatisation.

The SpeedVac dried samples underwent derivatisation using methyl chloroformate (MCF) (Smart et al., 2010), which makes the metabolites more volatile to enhance chromatographic separation of metabolites (Halket et al., 2005). The dried samples were then re-suspended in 400 µL 1 M sodium hydroxide with gentle vortex and transferred to labelled 8 mL silanised tubes (Thermo Scientific, USA) containing 334 µL methanol and 68 µL pyridine. Then, 40 µL MCF reagent (Sigma-Aldrich, cat. no. M3504) was added and vortexed for 30 s. Another 40 µL MCF reagent was added and vortexed for 30 s. After that, 400 µL chloroform was added and vortexed for ten seconds to extract the MCF derivatives from the mixture, followed by addition of 800 µL of 50 mM sodium bicarbonate solution and vortexed for ten seconds. The mixture was centrifuged (5810R, Eppendorf, Germany) at 6 °C, 2000 rpm (i.e., 845 rcf) for five minutes. The upper aqueous phase was removed, and a small amount of sodium sulphate was added to remove residual water. The chloroform phase with MCF derivatives was transferred into micro-inserts (Interlab, NZ) and placed in 1.5 mL amber GC vials (Interlab, NZ).

#### Sample analysis with GC-MS

The derivatised samples were analysed with a gas chromatograph (GC7890B, Agilent Technologies, USA) connected to a mass spectrometer with a quadrupole mass selective detector (MSD5977A, Agilent Technologies, USA) with 70 eV energy used in electron ionisation. For each measurement, 1 µL of the sample was injected with injector temperature at 290 °C in split-less mode, carried by helium gas with a constant flow of 1 mL min^− 1^ in a ZB-1701 capillary column (30 m × 250 μm (internal diameter) × 0.15 μm (film thickness), with 14% cyanopropyl phenyl, 86% dimethyl polysiloxane as stationary phase). Since mussels’ metabolites may include both polar, such as organic acids, and non-polar, such as fatty acids, substances, the use of this column may allow better separation of these metabolites for identification and analysis. The initial GC oven temperature was 45 °C and held for two minutes, then increased at a rate of 9 °C min^− 1^ until 180 °C, which was held for five minutes. Later, the temperature was further ramped at 40 °C min^− 1^ to 220 °C. After five minutes, the temperature increased at 40 °C min^− 1^ to 240 °C and was maintained for 11.5 min, followed by an increase at 40 °C min^− 1^ to 280 °C for ten minutes. The ion source temperature was 230 °C, and the quadrupole temperature was 150 °C. The mass spectrometer was operated in scan mode, starting after 4.52 min with a mass range of 38–550 m/z at four scans per second, and the detection threshold was set to 50 ion counts.

Different variants of quality control (QC) samples were also included to ensure the reproducibility of GC-MS measurements, as described by Nguyen (2020). These samples included pure chloroform solvent, non-derivatised alkane standard mixtures, derivatised amino and organic acids mixtures, blank samples and pooled samples from all tissue samples, which were co-derivatised with 20 µL 10 mM d_4_-Alanine. These QC samples were injected at the beginning of GC-MS analysis and intermittently after every 17 samples. All samples were injected randomly.

#### Spectral data processing

Raw mass spectrometry (MS) data were processed using Automated Mass Spectral Deconvolution and Identification System (AMDIS) software (NIST, USA) combined with an automated in-house R package “MassOmics” (University of Auckland, UoA; available online: https://github.com/MASHUOA/MassOmics) to identify metabolites in samples detected by GC-MS after MCF derivatisation (Smart et al., 2010). Metabolite identification and peak integration were performed using in-house MS for MCF derivatised standards constructed by UoA. Compounds identified were manually checked, and only those with ≥ 75% matched to the library based on their MS spectrum and respective retention time were retained for peak integration to obtain the abundance of each metabolite in terms of peak area. Metabolites with the peak integrated were batch filtered by “MassOmics” and individually checked with ChemStation (Agilent Technologies, USA) and AMDIS to remove laboratory contaminants. Finally, duplicate and abnormal records (based on total ID (i.e., how many times this compound has been measured in all samples), reference ion, retention time and peak area) were deleted. The cleaned data were normalised by the internal standard (i.e., d_4_-Alanine) followed by correction by the blank samples to remove any variations caused by sample handling processes in the laboratory. The blank-corrected data were then standardised by dry sample biomass.

### Measurement of haemolymph parameters

Haemolymph parameters, including pH, total antioxidant capacity (TAC) and osmolality, were measured. For TAC measurement, 50 µL of haemolymph was dispensed onto a disposable strip coupled with the portable assessment e-BQC device (Bioquochem S.L., Spain). Levels of TAC, expressed as µC, was the sum of slow- and fast-antioxidant responses (Delorme et al., 2021a, b). A Trolox (Sigma 238813) calibration curve (R^2^ = 0.97) was constructed by plotting the values obtained by the e-BQC device (µC) against different Trolox concentrations (0-1000 µM) in phosphate-buffered saline (pH = 7.42). The TAC values obtained from the device were then converted into Trolox equivalent antioxidant capacity (µM TEAC) with the calibration curve, to allow a more direct interpretation of antioxidant activity (Miller et al., 1993).

For measurement of haemolymph osmolality (mOsmol kg^− 1^), 10 µL of haemolymph was pipetted onto a filter paper disc (SS-033, EliTechGroup, USA), followed by automatic sample analysis using an osmometer (Vapro 5600, Wescor, USA), which was regularly calibrated against 100, 290 and 1000 mOsmol kg^− 1^ standards. Haemolymph pH was measured using a benchtop pH meter (Seven Compact, Mettler Toledo, USA), which was calibrated against AMP (2-aminopyridine, pH 6.776 at 25 °C) and Tris (Tris and KBr, pH = 8.096 at 25 °C) buffers (Ericson & Ragg, 2022). The osmolality and pH of the seawater to which mussels were exposed to before the experiment were also measured.

### Anaerobic enzyme activity

Enzyme activities of opine dehydrogenases, including strombine dehydrogenase (StDH) and alanopine dehydrogenase (AlDH) of the gills and adductor muscle tissues were analysed following the protocol described by Baldwin et al. (1992). In brief, samples (~ 0.1 g) were homogenised in an ice bath using an IKA T25 digital Ultra-turrax™ in 1 mL imidazole buffer (i.e., 1:10 weight/volume), which consisted of 50 mM imidazole-HCl (pH = 7), 2 mM MgCl_2_, 1mM disodium ethylenediaminetetraacetate dihydrate (Na-EDTA·2H_2_O) and 0.01% triton-X100. An extra mincing step was added for the adductor muscle before homogenisation. The homogenate was then transferred into a labelled 1.5 mL microcentrifuge tube, which was then centrifuged (Mikro 200R, Hettich, MA, USA) for 20 min at 4 °C and 12,000 rpm (i.e., 13684 rcf). The supernatant was pipetted into another 1.5 mL microcentrifuge tube and placed in a cold box on ice. For each sample, 10 µL of homogenate was pipetted into each of four wells of a 96-well flat-bottom microplate (#655101, Greiner, Austria). These four wells measured lactate dehydrogenase (LDH), StDH, AlDH; while the extra well was a control for background activity. The well for background activity control and the other three wells contained respectively 200 µL and 180 µL solution of imidazole buffer with 0.15 mM reduced nicotinamide adenine dinucleotide (NADH). For wells measuring StDH and AlDH, the solution also included 200 mM glycine and 100 mM L-alanine, respectively. Then, 20 µL of 2.5 mM pyruvate was added to these wells except to the background activity control. The final reaction volume for each well was 210 µL (Table S1). This was done in triplicate for each sample. Soon after pyruvate was added, the plates were read at 340 nm with a microtitre plate reader spectrophotometer (SpectraMax iD3, Molecular Devices, LLC., CA, USA) using kinetic mode. The measurements were read at 10-second intervals for eight minutes at 20 °C to record the changes in absorbance, which was then converted into specific enzyme activity expressed as international unit (i.u.; defined as the amount of enzyme to covert one µmol of NADH min^− 1^) after subtracting the LDH and background activities and normalising by biomass (i.e., i.u. g wet wt.^−1^) using the equation below:

$$Specific\, enzyme\, activity = \:\frac{\left|\text{s}\text{l}\text{o}\text{p}\text{e}\right|\times\:0.1608\times\:\frac{0.21}{0.01}\times\:\frac{1+\text{s}\text{a}\text{m}\text{p}\text{l}\text{e}\:\text{w}\text{t}.}{\text{s}\text{a}\text{m}\text{p}\text{l}\text{e}\:\text{w}\text{t}.}}{\text{s}\text{a}\text{m}\text{p}\text{l}\text{e}\:\text{w}\text{t}.}$$  

Where slope represents the decrease in absorbance over time (min), 0.1608 presents the changes in NADH converted into NAD^+^ per absorbance (calculated from 1/6.22, where 6.22 is the extinction coefficient for NADH (Klingenberg, 1974), 0.21 is the volume per well (i.e., reagents + sample; mL) and 0.01 is the sample volume (mL), and sample wt. represents the weight of the tissue sample used (g). Given the pathlength is 0.634 cm for 210 µL in a 96-well plate, the absorbance was, therefore, adjusted for the calculation of specific enzyme activity.

### Statistical analysis

#### Analysis of metabolomic data

The metabolomic data of the gill and adductor muscle were respectively normalised (i.e., autoscaling) before analysis. To identify those metabolites which were significantly different among treatments, one-way analysis of variance (ANOVA) was also conducted for each metabolite from control and post-harvest simulation groups (i.e., fixed factor), while considering the effects of different trials (i.e., random factor) using linear mixed effects model (LMM) from an R package “glmmTMB” (Brooks et al., 2017). It is important that the residuals satisfy the assumptions of normality and homogeneity of variance. For most metabolites analysed, the residuals of models exhibited homogeneous variance. For those that did not, the models were revised to account for residual heteroscedasticity by allowing residual variance to depend on a predictor (i.e., treatment). Models with non-normally distributed residuals remain robust despite assumption violations (Schielzeth et al., 2020). Partial least squares discriminant analysis (PLS-DA) was conducted to determine if there were any differences in metabolic profiles (i.e., clear separation) of mussels in the control and post-harvest groups, as well as obtaining the variable importance in projection (VIP) scores for each metabolite with R package mixOmics (Rohart et al., 2017). Metabolites that significantly differed (i.e., *p* < 0.05) between two groups (i.e., control and post-harvest) and with a VIP score > 1 were regarded as differentially expressed metabolites (DEMs) and used for the pathway analysis (*sensu* Xia et al., 2010).

Pathway analysis was performed using MetaboAnalyst 6.0 (available online: https://www.metaboanalyst.ca/) with DEMs data from the control and post-harvest groups mapping to the metabolic pathways of *Schistosoma mansoni* (flatworm), which has a closer phylogenetic relationship to molluscs (Adoutte et al., 2000), in the Kyoto Encyclopedia of Genes and Genomes (KEGG) database (Kanehisa & Goto, 2000), using all annotated molecules detected in this study (**Table S2**) as the reference set of compounds (Wieder et al., 2021). Pathways involving more than two annotated metabolites (i.e., DEMs) that matched the KEGG database with *p* value < 0.05, false discovery rate < 0.1 and impact score > 0.1 were regarded as significantly enriched pathways of interest.

To test whether the metabolic profiles of mussels varied among the treatments, permutation multivariate analysis of variance (PERMANOVA; Anderson, 2001) with 500 permutations using an R package “BiodiversityR” (Kindt & Coe, 2005) were conducted in gill and adductor muscle metabolomic data to isolate the differences in metabolic profiles of mussels exposed to five treatments (i.e., control, post-harvest, MgCl_2_ immersion, 14 °C and 4 °C; fixed factor) after considering the effects of trials (i.e., random factor). Then, one-way ANOVA was conducted to compare each metabolite among the five conditions to select significantly different metabolites. Heatmaps with hierarchical clustering were constructed using the R package “pheatmap” (Kolde, 2019) to visualize the selected metabolites. Hierarchical clustering was used to generate dendrograms, which were integrated with the heatmaps to depict the similarity among metabolites while also displaying their concentrations across different treatments.

#### Analysis of haemolymph parameters

Haemolymph parameters (i.e., TAC, pH and osmolality) of mussels exposed to the five treatments (see previous section) were analysed with one-way ANOVA using LMM to illustrate the variations among exposure conditions (i.e., fixed factor) while considering the effects of different trials (i.e., random factor). Since the osmolality of seawater/solution (i.e., external osmolality) of different treatments varied (~ 800–1000 mOsmol kg^− 1^), which would potentially have effects on mussel haemolymph osmolality, the external osmolality of different treatments was included in the model as a covariate for the analysis of haemolymph osmolality. Post hoc multiple comparisons were conducted using an R package “emmeans” (Lenth, 2022) to detect the differences in haemolymph parameters with Benjamini-Hochberg adjustment of *p* values to account for type 1 error.

#### Analysis of anaerobic enzyme activities

Activity levels of lactate dehydrogenase (LDH), strombine dehydrogenase (StDH) and alanopine dehydrogenase (AlDH) from the five treatments (i.e., control, post-harvest, MgCl_2_ immersion, 14 °C and 4 °C; fixed factor) were analysed with LMM from the “glmmTMB” package with trial as a random factor (see 2.8.1). Once the enzyme activities were significantly impacted by the treatments, pairwise comparisons with the adjustment of *p* values were conducted using the “emmeans” package (see 2.8.2).

## Results

### Effects of post-harvest handling on mussels

#### Metabolic profile

**Fig. 2:**
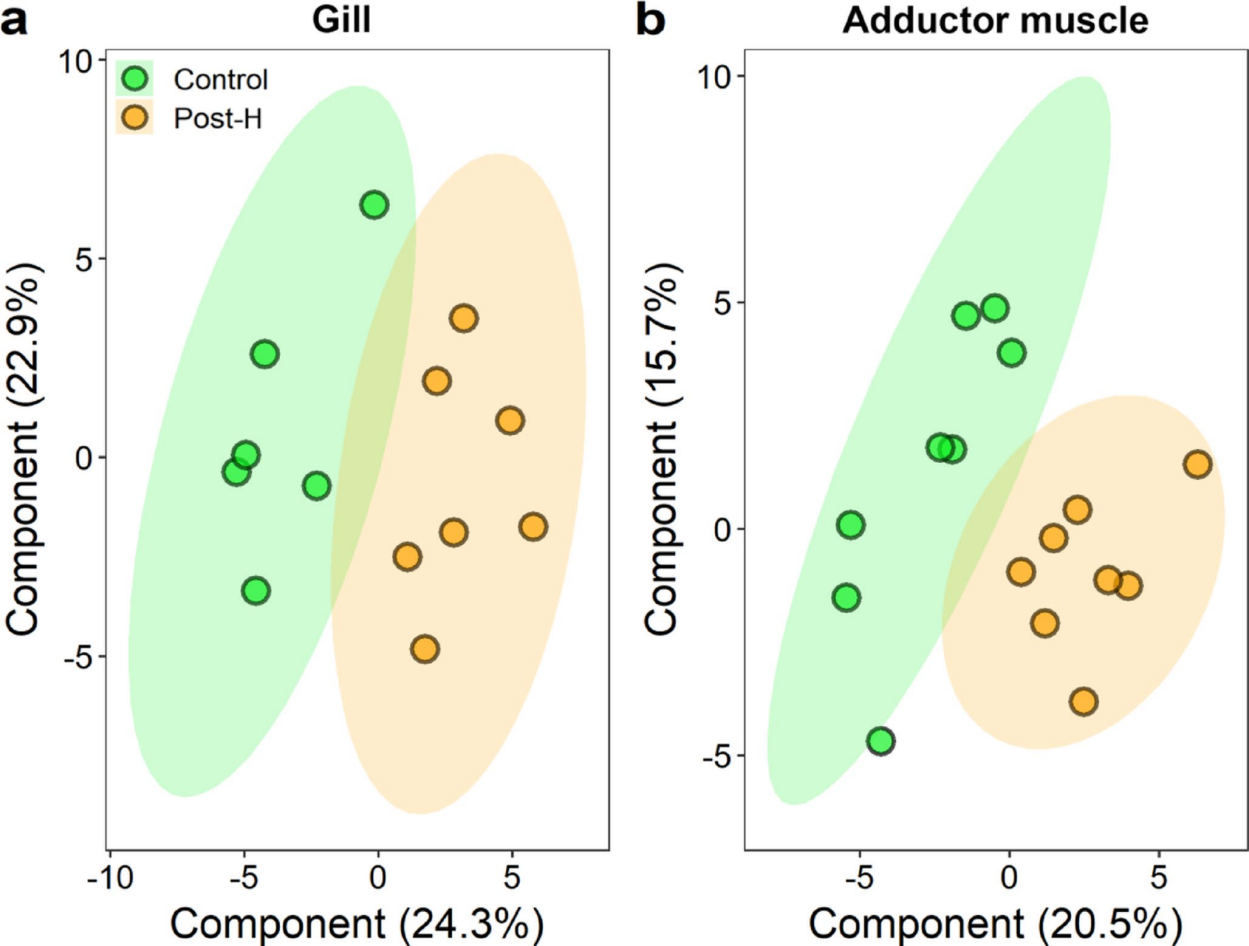
PLS-DA plots depicting the differences in metabolic profiles in (a) gills and (b) adductor muscles from control (green) and post-harvest (Post-H) simulated (orange) mussels. The shaded areas on the plots represented 95% confidence ellipses

Metabolomic analysis of gills and adductor muscles demonstrated the difference between metabolite profiles of control and post-harvest simulated mussels, reflected in PLS-DA plots showing clear separation of mussels from the two groups (Fig. 2). There were 21 and 14 DEM respectively identified in gill and adductor muscle samples (one-way ANOVA, *p* < 0.05 and VIP scores > 1; Table S3). Also, the metabolic pathway analysis identified 17 and 14 metabolic pathways in the present study for gills and adductor muscle, respectively (Table S4). Three pathways involved in amino acid and sugar metabolism were identified as affected primary metabolic pathways of interest for both gill and adductor muscle samples due to significantly enriched metabolites associated with these pathways (i.e., *p* values < 0.05, FDRs < 0.1 and impact values > 0.1) in relation to the effects of post-harvest treatment (Table 2). For gill samples, the citric acid cycle (CAC) pathway is essential for aerobic metabolism. With an impact score close to 0.1 (i.e., 0.077), it was also regarded as a key pathway of interest in this study.

**Table 2:** List of significantly enriched metabolic pathways of interest in mussel (a) gills and (b) adductor muscle after post-harvest simulation

Pathway	Detected enriched metabolites	Hits	-log(*p*)	FDR	Impact	Enrichment ratio
*(a) Gill*						
Citrate cycle (CC)	Succinic acid, malic acid, phosphoenolpyruvic acid	3 [6]	4.698	< 0.001	0.077	6.778
Alanine, aspartate and glutamate metabolism (AAG)	Aspartic acid, Alanine, Succinic acid	3 [6]	4.114	< 0.001	0.196	7.564
Glycolysis / Gluconeogenesis (GG)	Phosphoenolpyruvic acid	1 [1]	3.644	0.001	0.101	8.685
Tryptophan metabolism (TPM)	Tryptophan	1 [1]	1.946	0.028	0.235	5.471
*(b) Adductor muscle*						
Alanine, aspartate and glutamate metabolism (AAG)	Aspartic acid, Alanine, Succinic acid	3 [1]	4.098	0.001	0.196	8.970
Tyrosine metabolism (TM)	Tyrosine	1 [2]	1.590	0.051	0.391	5.326
Cysteine and methionine metabolism (CMM)	Methionine, Cysteine	2 [5]	1.477	0.058	0.323	3.216

The abbreviation for each metabolic pathway is in parentheses. Numbers in the square brackets represent the total number of compounds involved in the metabolic pathways detected in this study

### Subsequent immersion in different potential stress-relieving conditions

#### Metabolic profiles

**Fig. 3:**
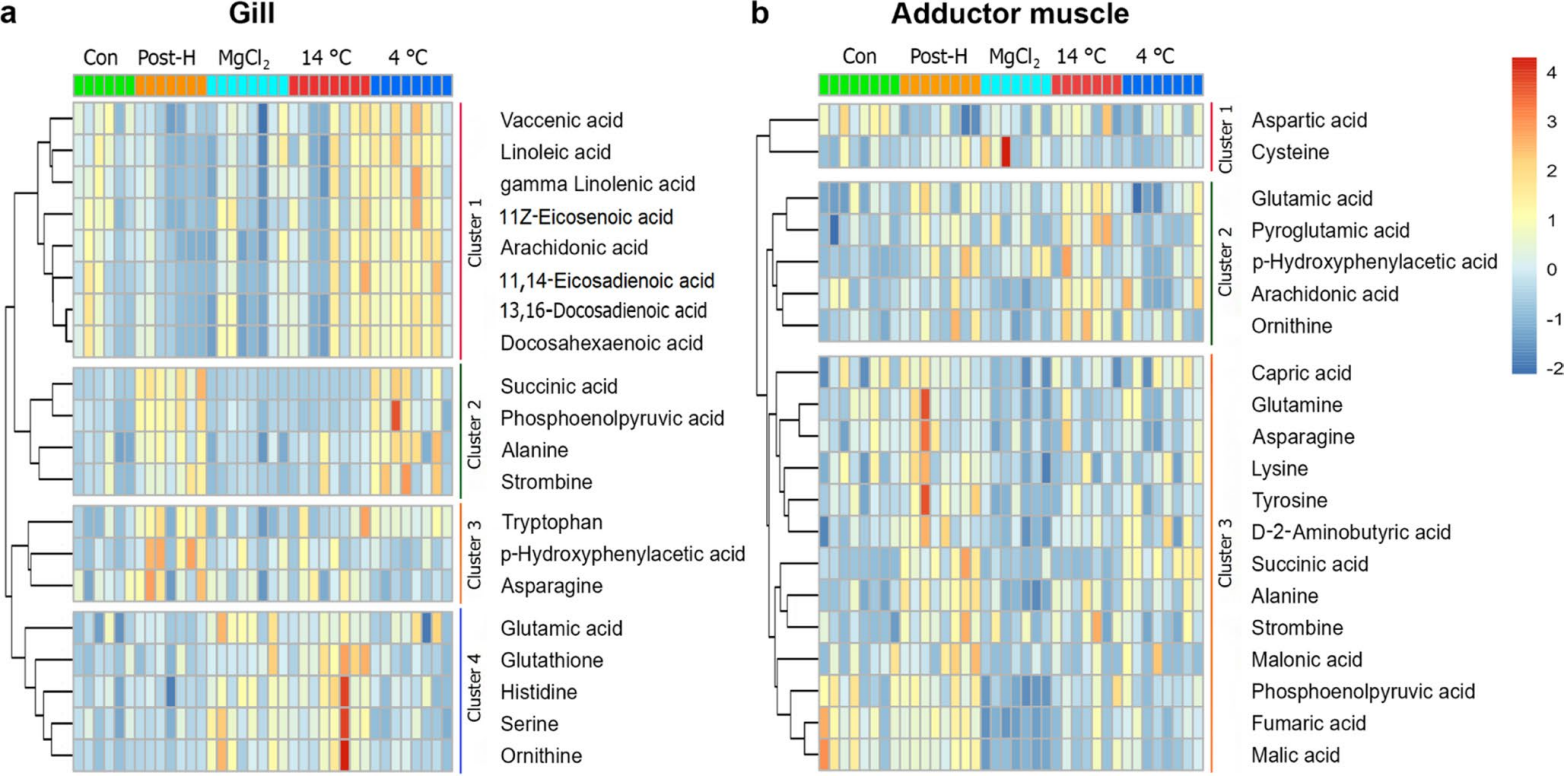
Heatmaps showing the top 20 metabolites that differed significantly (*p* < 0.05) in (a) gills and (b) adductor muscles among treatments (Con = control, Post-H = mussels after post-harvest simulation, MgCl_2_ = mussels after two-hour MgCl_2_ immersion, and 14 °C and 4 °C = mussels after two-hour immersion in 14 °C and 4 °C seawater, respectively). Columns represent replicate samples and each row represents an individual metabolite. The colour varied from dark red to deep blue representing high to low values of metabolite concentrations. Metabolites were grouped into four clusters in gills and three clusters in adductor muscles, which are involved in energy metabolism, amino and fatty acids metabolism, and cellular stress response

PERMANOVA showed that there were significant differences (*p* < 0.01, Table 3) in metabolic profiles of gill and adductor muscle of mussels in control and post-harvest treatments. One-way ANOVA detected 33 and 21 metabolites with significantly different enrichment levels (*p* < 0.05) among treatments for gill and adductor muscle samples, respectively (**Table S5**). Heatmaps showed the relative abundance of the top 20 metabolites that significantly differed among treatments in gills and adductor muscle tissue samples, which could be grouped into three clusters (Fig. 3). For gill samples, the metabolites in Cluster 1 decreased during post-harvest simulation and increased during hypothermic shock (i.e., 4 °C). Metabolites in Cluster 2 increased during post-harvest simulation, and maintained lower levels in mussels from control groups, and 14 °C and MgCl_2_ immersion treatments. The levels of metabolites in Cluster 3 increased during post-harvest simulation. Abundance of metabolite in Cluster 4 were higher in mussels after immersion in 14 °C seawater. (Fig. 3a). For adductor muscle samples, the metabolite abundance of Cluster 1 decreased during post-harvest simulation. In contrast, metabolite abundance of Cluster 2 was higher in mussels after post-harvest simulation and 14 °C immersion. Metabolite levels of Cluster 3 increased during post-harvest simulation and remained at lower levels in mussels from other treatment groups (Fig. 3b).

**Table 3:** PERMANOVA to compare the variation of metabolic profiles of (a) gill and (b) adductor muscles sampled from mussels after different treatments (i.e., control, post-harvest, immersion in 14 °C, 4 °C and MgCl_2_ conditions) nesting trials at significance level 0.05

Source	df	MS	Pseudo-F	*p* value
(a) Gill				
Treatment	4	130.90	2.24	**0.002**
Residual	30	56.65		
(b) Adductor muscle				
Treatment	4	99.49	1.63	**0.002**
Residual	31	60.81		

Significant *p* values were in bold

**Fig. 4:**
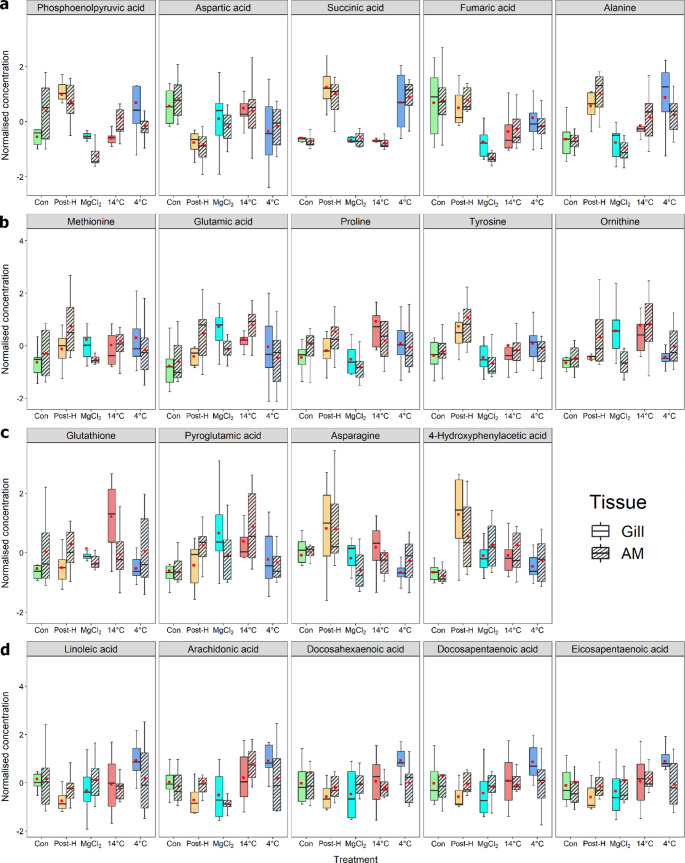
Boxplots showing variations of key metabolites involved in (a) energy metabolism (top panel), (b) amino acid metabolism (second panel), (c) cellular stress responses (third panel) and (d) lipid/fatty acid metabolism (bottom panel) of gill (open) and adductor muscle (stripped) of mussels after different treatments (Con = control, Post-H = mussels after post-harvest simulation, MgCl_2_ = mussels after two-hour MgCl_2_ immersion, and 14 °C and 4 °C = mussels after two-hour immersion in 14 °C and 4 °C seawater, respectively). The red square represents the mean

The metabolites, which differed significantly among treatments, were related to energy metabolism, amino acid metabolism, cellular stress response (i.e., antioxidant activity) and fatty acid metabolism. Mussels after two-hour post-harvest simulation had increased levels of end products of anaerobic metabolism (e.g., succinate and alanine) and amino acids (e.g., aspartic acid and ornithine), and decreased levels of polyunsaturated fatty acids (PUFA). Although the levels of such compounds generally returned to those of control mussels during subsequent re-immersion, the metabolomic responses (i.e., metabolites for energy and fatty acid metabolism) of mussels differed among re-immersion treatments (Fig. 4). With re-immersion at 4 °C seawater, mussels had relatively higher levels of succinate, alanine and PUFA.

#### Haemolymph parameters

**Fig. 5:**
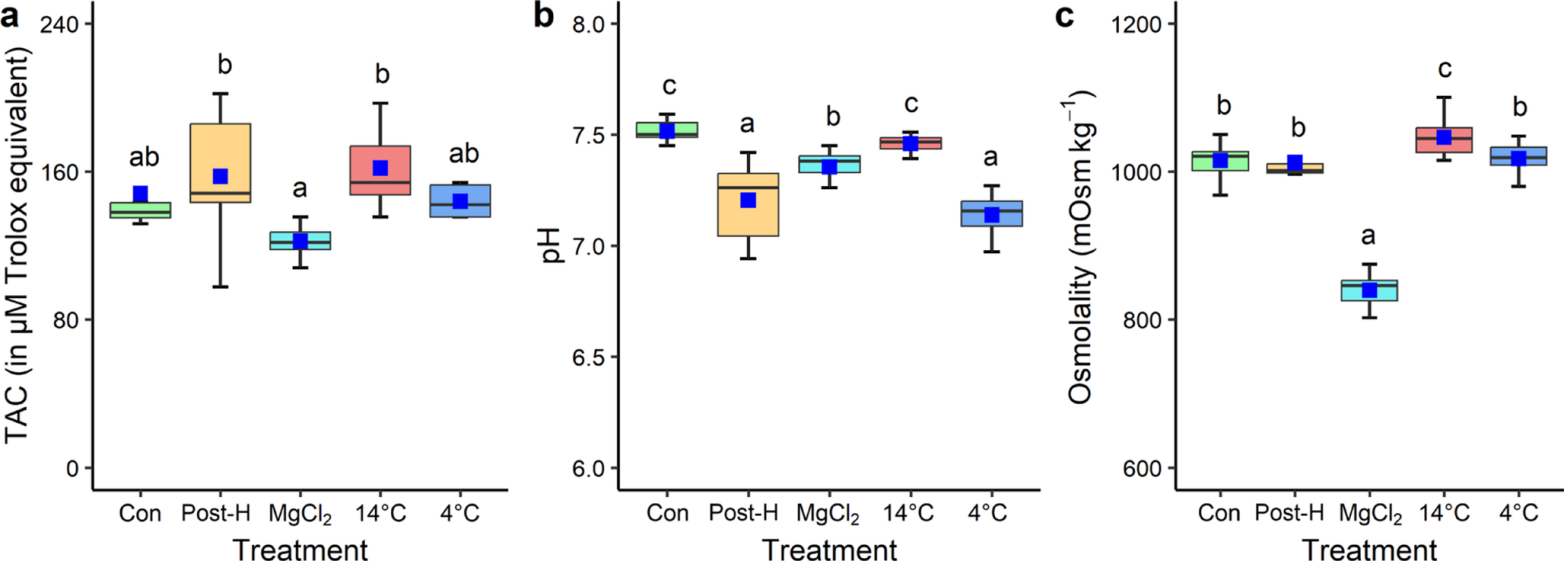
Boxplots depicting the variation of (a) total antioxidant capacity (TAC), (b) pH and (c) osmolality in haemolymph samples of mussels from different treatments (Con = control, Post-H = mussels after post-harvest simulation, MgCl_2_ = mussels after two-hour MgCl_2_ immersion, and 14 °C and 4 °C = mussels after two-hour immersion in 14 °C and 4 °C seawater, respectively). Different letters represent significant differences among treatments

Total antioxidant capacity (TAC) of mussel haemolymph varied significantly among mussels exposed to post-harvest simulation and two-hour immersion in 14 °C seawater, with higher and more variable TAC compared to other treatments (Fig. 5; *χ*^2^_4_ = 14.64, *p* < 0.01). Similarly, haemolymph pH differed significantly among treatments. Post-harvest mussels and mussels following two-hour immersion in 4 °C seawater had significantly lower pH than mussels at 14 °C and MgCl_2_ treatments (*χ*^2^_4_ = 111.33, *p* < 0.001). Haemolymph osmolality was also significantly different among treatments with mussels after two-hour 40 g L^− 1^ MgCl_2_ immersion having lower osmolality than other treatments (*χ*^2^_4_ = 24.21, *p* < 0.001).

#### Anaerobic enzymes

**Fig. 6:**
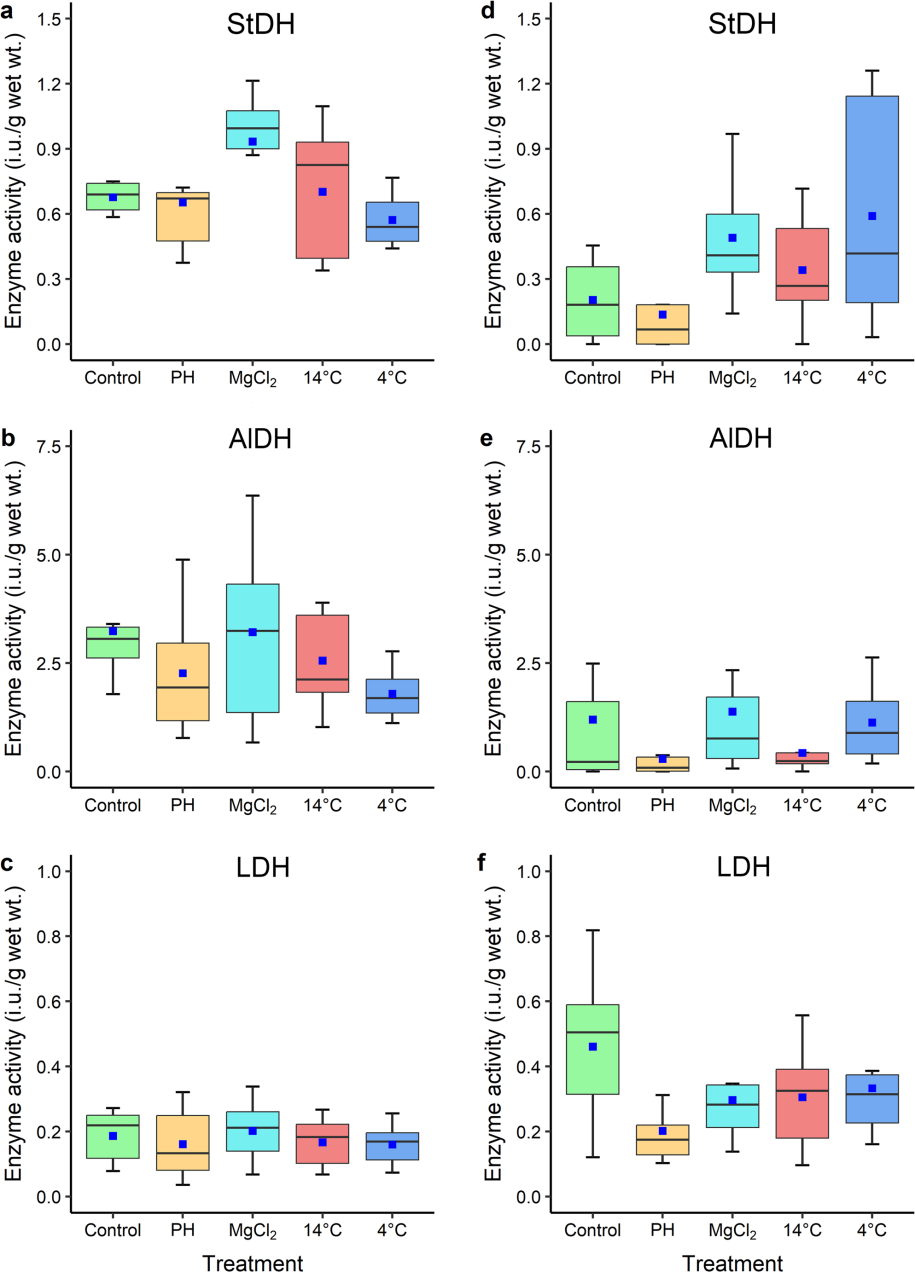
Boxplots showing the variation of anaerobic enzyme activity levels (mass-normalised international unit, i.u.) of (a, d) StDH, (b, e) AlDH and (c, f) LDH, of gills (left panel) and adductor muscles (right panel) of mussels from different treatments (Con = control, Post-H = mussels after post-harvest simulation, MgCl_2_ = mussels after two-hour MgCl_2_ immersion, and 14 °C and 4 °C = mussels after two-hour immersion in 14 °C and 4 °C seawater, respectively)

There were tissue-specific differences in anaerobic enzyme responses to the treatments with no significant difference in specific activity levels of StDH in gills (*χ*^2^_4_ = 8.61, *p* = 0.071); but strong differences between adductor muscles (*χ*^2^_4_ = 14.72, *p* = 0.005) among treatments (Fig. 6a, d). For AlDH there were significant differences in activity between gills (*χ*^*2*^_4_ = 11.33, *p* = 0.021) and adductor muscles (*χ*^2^_4_ = 11.46, *p* = 0.022) among treatments (Fig. 6b, e). Furthermore, LDH activity levels were only different in adductor muscles (*χ*^2^_4_ = 11.59, *p* = 0.021) but not in gills (*χ*^2^_4_ = 1.67, *p* = 0.796) after treatment exposure (Fig. 6c, f). Subsequent pairwise comparisons, however, did not differentiate the treatments that significantly impacted the specific activity levels of enzymes. Generally, gills had higher levels of LDH and AlDH activities than adductor muscles, whereas adductor muscles had higher StDH activity levels than gills. Also, re-immersed mussels generally had higher anaerobic enzyme activity regardless of tissues.

## Discussion

### Effects of post-harvest simulation on metabolomics of *P. canaliculus*

Post-harvest aerial exposure treatment triggers the anaerobic pathways of mussels to undergo anaerobic breakdown of glucose to form ATP due to a lack of adequate oxygen supply, resulting in the accumulation of anaerobic end products (Collicutt & Hochachka, 1977). In the present study, mussels after two-hour post-harvest simulation increased succinic acid, alanine and strombine levels, as well as decreased aspartic acid levels in gills and adductor muscle. This may suggest that *P. canaliculus* utilised glucose-succinate coupling with aspartate-succinate and glucose-opine pathways to simultaneously ferment glycogen and aspartate for anaerobic ATP production to generate energy dealing with stress induced by post-harvest simulation. The accumulation of alanine, which is converted from pyruvic acid, also helps fix ammonium in the absence of water (Zurburg & De Zwaan, 1981), preventing its toxic effects until excretion is possible. Thus, this serves as a crucial adaptation for managing hypoxic stress during aerial exposure in post-harvest simulation. Such patterns between alanine and succinic acid, and aspartic acid were observed in bivalves such as *Limaria* (formerly *Lima*) *hians*, *Cerastoderma* (formerly *Cardium*) *edule*, and *Mytilus galloprovincialis* during anoxia (Gäde, 1983b; Isani et al., 1995; Meinardus & Gäde, 1981). The imbalance between increased ATP hydrolysis relative to synthesis results in elevated proton release alongside anaerobic metabolites, such as succinic acid, together with accumulating CO_2_ and carbonic acid within tissues and fluids due to impaired gas exchange in the air (Pörtner et al., 1984; Sukhotin & Pörtner, 1999), leading to a decrease in cellular pH in the mussels, which was also recorded in other mussels such as *Mytilus edulis* and *M. galloprovincialis* (Anestis et al., 2010; Booth et al., 1984).

### Metabolic and haematological responses of mussels in different treatments

In this study, post-harvest mussels underwent anaerobiosis as indicated by increased levels of succinic acid and alanine, and decreased levels of aspartic acid, which are respectively the key end products and substrates of anaerobic pathways (see Harcet et al., 2013 for review). The post-harvest mussels may also be subject to osmotic stress during associated aerial exposure as there were increased levels of free amino acids (FAA), such as alanine, proline, tyrosine, tryptophan, methionine and asparagine, which may act as osmolytes that maintain the intracellular osmotic pressure for osmoregulation (Deaton et al., 1984; Zurburg & De Zwaan, 1981). Immersing post-harvest mussels in 14 °C or MgCl_2_ treatments could alleviate emersion-induced stress, as the treated mussels showed decreased end-products and increased levels of substrate for anaerobic pathways. Meanwhile levels of FAA of post-harvest mussels after re-immersion, particularly in MgCl_2_ condition, returned to levels similar to those of control mussels, suggesting that the potential osmotic stress could be mitigated in these treatments. Low temperatures and anaesthetics have been shown to effectively alleviate the effects of handling stress in other bivalves (e.g., Anacleto et al., 2013; Heasman et al., 1995; Suquet et al., 2009). Re-immersion at 4 °C could, however, impose a cold shock on the mussels, which may divert energy from anaerobic metabolism to deal with the stress induced by sudden temperature drop, as recorded in *M. galloprovincialis* (Wang et al., 2018).

Furthermore, aerial exposure may impose oxidative stress on mussels (e.g., De Almeida et al., 2005; Moreira et al., 2021; Wang et al., 2018) through the production of reactive oxygen species (ROS). This may trigger the antioxidative responses to protect mussel tissues from further damage by ROS (Delorme et al., 2021a, b, 2024). In this study, post-harvest mussels had increased levels of 4-hydroxyphenylacetic acid and tyrosine. These have antioxidant activities (Biskup et al., 2013; Gülçin, 2007; Lewinska et al., 2007) and subsequently decreased during re-immersion. Such antioxidants may support oxidative stress management during post-harvest simulation. Conversely, increased levels of another important antioxidant, glutathione (Meister, 1989), following 14 °C re-immersion compared to post-harvest mussels, was consistent with the inference that glutathione plays a key role in removing excessive ROS produced during re-oxygenation (Hermes-Lima et al., 2015). This may imply greater oxidative stress was present in mussels during post-harvest simulation and 14 °C re-immersion. In contrast, total antioxidant capacity (TAC) of mussels immersed in MgCl_2_ and 4 °C treatments returned to a level similar to control. Altogether, changes in levels of individual antioxidants are likely to elicit variations of TAC in mussels among the treatments.

Fatty acids (FA), which are the building blocks of complex lipids, are essential for the health and survival of organisms, including marine species (Zhukova, 2019). Since polyunsaturated fatty acids (PUFA) are a major constituent of lipids in *P. canaliculus* (Miller & Tian, 2018), a decrease in PUFA of mussels may indicate the depletion of its energy storage to cope with the changing environment caused by post-harvest handling (Venter et al., 2021). Alternatively, decreased PUFA levels in post-harvest mussels could also reflect an increasing efficacy of cellular defence against oxidative stress associated with hypoxia during emersion, mitigating damage to the cell membranes of bivalves (e.g., Jing et al., 2023). A reduction in PUFA levels, which are prone to oxidation, can help protect cell membranes from oxidative stress (Crockett, 2008), thereby preserving mussel membrane integrity during post-harvest simulation. Low temperatures could, however, decrease cell membrane fluidity due to increased lipid packing order, which decreases the membrane permeability and disrupts transmembrane enzyme activities and ion transport (Hochachka & Somero, 2002; Yoon et al., 2022). At decreasing temperatures, the proportion of PUFA in lipids could increase to maintain the cell membrane fluidity and, thus, the permeability (Logue et al., 2000). In this study, *P. canaliculus* re-immersed in 4 °C seawater had the highest PUFA levels among treatments, which indicates re-modelling of cellular membranes by increasing the PUFA content in response to the cold environment, as shown in the cold-tolerant mussel, *Mytilus edulis* (Fokina et al., 2018). Levels of PUFA, therefore, varied in different conditions reflecting their multifunctional properties, serving as an energy source and cellular defence.

### Anaerobic enzyme activity

Aerial exposure-mediated hypoxia could induce anaerobic glycolysis in organisms. Alternative glycolytic metabolic pathways engaging in the production of opines have been observed in molluscs during anaerobiosis (Harcet et al., 2013). A significant increase in opine dehydrogenase activity in the adductor muscles of *P. canaliculus* was observed after four-day aerial exposure, according to previous studies (Esene, 2012; Powell, 2016). In the present study, two-hour aerial exposure associated with post-harvest simulation did not increase all tested anaerobic enzymes of LDH, StDH and AlDH in the gills and adductor muscles of *P. canaliculus*, which likely suggests such exposure duration was not sufficient to stimulate the synthesis and (or) activation of opine dehydrogenases. Re-immersion, however, generally increased anaerobic enzyme activity levels of *P. canaliculus*. This may imply that mussels still undergo anaerobic metabolism to compensate for the energy deficit due to hypoxic stress associated with post-harvest simulation during re-immersion (De Zwaan et al., 1983). Tissue-specific differences in the distribution of the anaerobic enzymes have been observed in various marine molluscs. For example, the mussel, *Mytilus edulis* had more AlDH in gills than in the adductor muscles, while StDH was higher in the adductor muscles (Dando et al., 1981). We report a similar pattern for *P. canaliculus* in this present study. In bivalves, muscle tissues need to deal with multiple low-oxygen stressors, including environmental hypoxia that arises from aerial exposure and functional hypoxia due to muscle work (e.g., valve closure), which could promote the activity level of StDH for anaerobic energy production during elevated energy demand (Nicchitta & Ellington, 1984). Bivalve gills, which represent critical diffusion surfaces, are metabolically active and have close contact with the external environment and, thus, are more susceptible to environmental stress. The generally higher activity level of AlDH could ensure the maintenance of ATP production and cellular redox state to cope with (e.g., osmotic and oxidative) stress to minimise their damage (Dando et al., 1981; Korycan & Storey, 1983).

## Conclusions

This study demonstrated the differential biochemical and haematological responses of the green-lipped mussel, *Perna canaliculus*, after short-term aerial exposure as a mimic of post-harvest handling and subsequent re-immersion into different treatments being surveyed for their potential to alleviate post-harvest stress. After post-harvest simulation, the metabolic pathways for anaerobic energy production and cellular stress responses in both gills and adductor muscles were upregulated, along with markers of acidosis, increased oxidative responses and compromised nutritional status, as indicated by reduced unsaturated fatty acid content. Although re-immersion in 14 °C seawater could help mussels recover from anaerobiosis, it was also accompanied by greater oxidative stress. While immersion in 4 °C water could restore mussel fatty acid content, it appears individuals are not able to recover from anaerobiosis during the immersion process. Mussels that received the MgCl_2_ treatment had reduced anaerobiosis, had less oxidative stress, and had nutritional status markers similar to those of the control, suggesting a better condition to ameliorate the stress associated with the post-harvest condition, which could maximise mussel quality and shelf-life when exported live. Given the significant volume of commercial mussels processed daily, liquid-based treatments are logistically challenging. Future research should focus on optimising the implementation of this treatment while assessing recovery times to minimise its potential effects on meat yield, texture, and toughness.

## Electronic supplementary material

Below is the link to the electronic supplementary material.

Supplementary Material 1

## Data Availability

The data in this study will be available on reasonable request after permission has been granted from the corresponding author.
