# Supplementary material for: Metabolomic and physiological insights to ameliorate post-harvest stress in cultured mussels
Source: Metabolomics. 2025 Jun 26;21(4):91. doi: 10.1007/s11306-025-02289-1 (PMC12202636; doi:10.1007/s11306-025-02289-1)
Supplement: Supplementary file 1 — Supplementary Material 1 [file 11306_2025_2289_MOESM1_ESM.docx]

**Metabolomic and physiological insights to ameliorate post-harvest stress in cultured mussels**

Cheng, M.C.F.^1,2*^, Welford, M.R.V.^1,2^, Zamora, L.N.^1,2^, Delorme, N.J.^2^, Ragg, N.L.C.^2^, Hickey, A.J.R. ^1^, Dunphy, B.J. ^1^

^1^ School of Biological Sciences, University of Auckland, Private Bag 92019, Auckland, 1142, New Zealand

^2^ Cawthron Institute, Private Bag 2, Nelson 7042, New Zealand

*Email of corresponding author: [cche448@aucklanduni.ac.nz](mailto:cche448@aucklanduni.ac.nz) (M.C.F. Cheng)

Email: [b.dunphy@auckland.ac.nz](mailto:b.dunphy@auckland.ac.nz) (B.J. Dunphy)

**Supporting Information**

This supporting information contains relevant tables cited in the main text.

Fig. S1 Comparison of different volume of treatment solutions (i.e., 40 g L^-1^ MgCl_2_) on mussel heart rates.


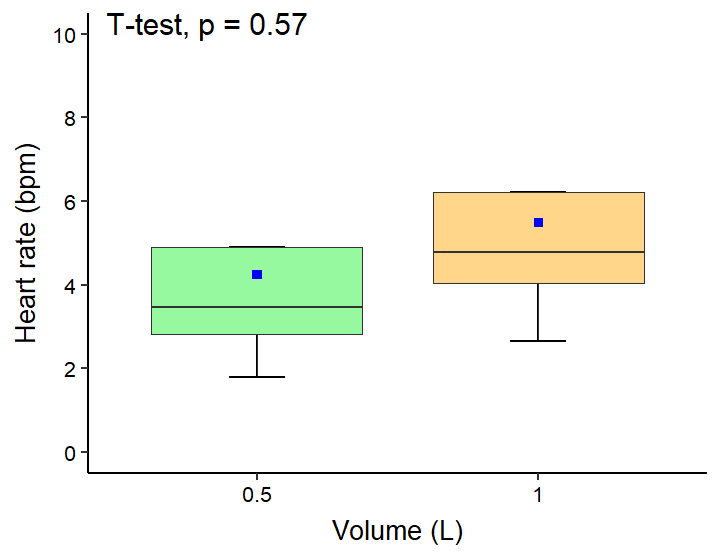


Fig. S2 Comparison of different volume of treatment solutions (i.e., 40 g L^-1^ MgCl_2_) on mussel haemolymph total antioxidant capacity (TAC), pH and osmolality. Data of TAC and osmolality did not pass normality test and non-parametric Mann-Whitney test was conducted.


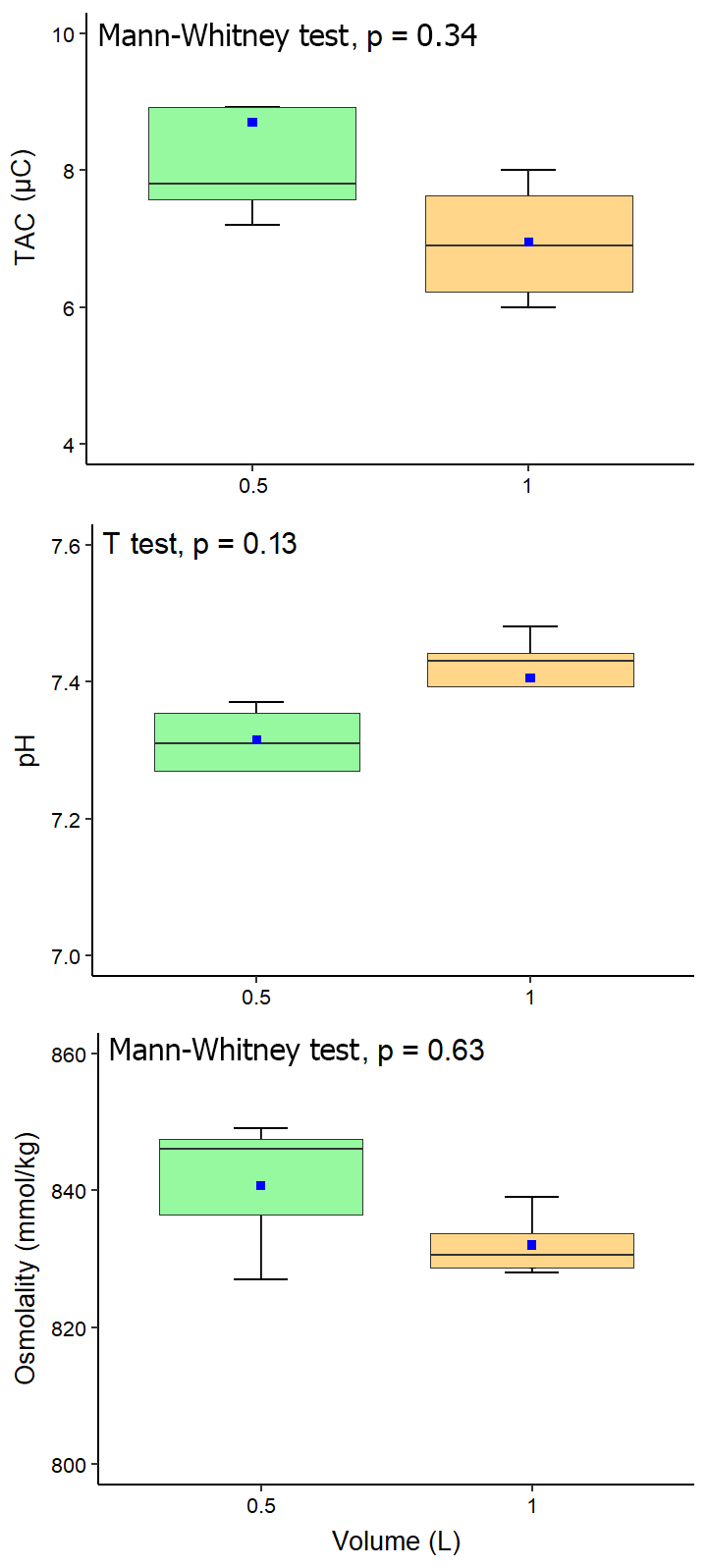


**Table S1** Composition of solutions for each sample used for measurement of background activities (i.e., Control), lactate dehydrogenase (LDH), strombine dehydrogenase (SDH) and alanopine dehydrogenase (AlDH) in spectrophotometry.

|  | Control | LDH | SDH | AlDH |
| --- | --- | --- | --- | --- |
| Sample | 10 µl | 10 µl | 10 µl | 10 µl |
| Solution | 200 µl  (imidozale buffer, 0.15 mM NADH) | 180 µl  (imidozale buffer, 0.15 mM NADH) | 180 µl  (imidozale buffer, 0.15 mM NADH, 200 mM glycine) | 180 µl  (imidozale buffer, 0.15 mM NADH, 100 mM L-alanine) |
| Pyruvate (2.5 mM) | 0 µl | 20 µl | 20 µl | 20 µl |
| Total volume | 210 µl | 210 µl | 210 µl | 210 µl |

**Table S2** The metabolites detected in the mussel tissues in the present study, which were used as reference library for pathway analysis (AAD = Amino acid derivative, AAMI = Amino acid metabolic intermediates, NAA = Non-standard amino acid).

| **Category** | **Metabolite** | **KEGG ID** | **Category** | **Metabolite** | **KEGG ID** | **Category** | **Metabolite** | **KEGG ID** |
| --- | --- | --- | --- | --- | --- | --- | --- | --- |
| Amino acid | Cysteine | C00097 | NAA | gamma-Aminobutyric acid | C00334 | Fatty acid | Docosahexaenoic acid | C06429 |
| Amino acid | Lysine | C00047 | AAD | Glutathione | C00051 | Fatty acid | Palmitic acid | C00249 |
| Amino acid | Glycine | C00037 | AAD | Pyroglutamic acid | C01879 | Fatty acid | Vaccenic acid | C08367 |
| Amino acid | Homocysteine | C00155 | AAD | 4-Hydroxyphenylacetic acid | C00642 | Fatty acid | Myristic acid | C06424 |
| Amino acid | Glutamic acid | C00025 | AAMI | 3-Methyl-2-oxovaleric acid | C00671 | Fatty acid | Linoleic acid | C01595 |
| Amino acid | Aspartic acid | C00049 | AAMI | 3-Aminoisobutanoic acid | C05145 | Organic acid | Malonic acid | C00383 |
| Amino acid | Histidine | C00135 | AAMI | Aminoadipic acid | C00956 | Organic acid | Fumaric acid | C00122 |
| Amino acid | Glutamine | C00064 | AAMI | Cystathionine | C02291 | Organic acid | Glyoxylic acid | C00048 |
| Amino acid | Methionine | C00073 | Imino acid | Strombine | C03790 | Organic acid | Itaconic acid | C00490 |
| Amino acid | Alanine | C00041 | Fatty acid | Pentadecanoic acid | C16537 | Organic acid | Citric acid | C00158 |
| Amino acid | Asparagine | C00152 | Fatty acid | 11,14-Eicosadienoic acid | C16525 | Organic acid | Glutaric acid | C00489 |
| Amino acid | Proline | C00148 | Fatty acid | Capric acid | C01571 | Organic acid | Succinic acid | C00042 |
| Amino acid | D-2-Aminobutyric acid | C02261 | Fatty acid | Dodecanoic acid | C02679 | Organic acid | Phosphoenolpyruvic acid | C00074 |
| Amino acid | *cis*-4-Hydroxyproline | C01157 | Fatty acid | Eicosapentaenoic acid | C06428 | Organic acid | *cis*-Aconitic acid | C00417 |
| Amino acid | L-Tryptophan | C00078 | Fatty acid | 11Z-Eicosenoic acid | C16526 | Organic acid | Nicotinic acid | C00253 |
| Amino acid | beta-Alanine | C00099 | Fatty acid | alpha-Linolenic acid | C06427 | Organic acid | Malic acid | C00149 |
| Amino acid | Leucine | C00123 | Fatty acid | Docosadienoate (22:2n6) | C16533 | Organic acid | 2-Hydroxybutyric acid | C05984 |
| Amino acid | Ornithine | C00077 | Fatty acid | 9E-Heptadecenoic acid | C16536 |  |  |  |
| Amino acid | Phenylalanine | C00079 | Fatty acid | Adrenic acid | C16527 |  |  |  |
| Amino acid | Tyrosine | C00082 | Fatty acid | Docosapentaenoic acid  (22n-3) | C16513 |  |  |  |
| Amino acid | Valine | C00183 | Fatty acid | Arachidonic acid | C00219 |  |  |  |
| Amino acid | Threonine | C00188 | Fatty acid | gamma-Linolenic acid | C06426 |  |  |  |
| Amino acid | Serine | C00065 | Fatty acid | Stearic acid | C01530 |  |  |  |

**Table S3** Differentially expressed metabolites from (a) gills and (b) adductor muscle samples of control and post-harvest simulated mussels for pathway analysis (VIP: Variable importance in projection obtained from PLS-DA plot).

| (a) Gill |  |  |  |  | (b) Adductor muscle |  |  |  |  |
| --- | --- | --- | --- | --- | --- | --- | --- | --- | --- |
| Metabolite | *χ*^2^ | *df* | *p* value | VIP | Metabolite | *χ*^2^ | *df* | *p* value | VIP |
| Succinic acid | 42.329 | 1 | 7.720‧10^-11^ | 2.033 | Aspartic acid | 34.192 | 1 | 4.990‧10^-9^ | 2.185 |
| Phosphoenolpyruvic acid | 34.063 | 1 | 5.340‧10^-9^ | 1.977 | Alanine | 31.197 | 1 | 2.330‧10^-8^ | 2.300 |
| Aspartic acid | 20.767 | 1 | 5.186‧10^-6^ | 1.820 | Succinic acid | 29.487 | 1 | 5.630‧10^-8^ | 2.344 |
| Strombine | 16.219 | 1 | 5.640‧10^-5^ | 1.481 | Strombine | 13.485 | 1 | 2.404‧10^-4^ | 1.992 |
| Alanine | 13.655 | 1 | 2.196‧10^-4^ | 1.663 | 4-Hydroxyphenylacetic acid | 11.913 | 1 | 5.574‧10^-4^ | 1.868 |
| 4-Hydroxyphenylacetic acid | 15.017 | 1 | 1.066‧10^-4^ | 1.647 | 9E-Heptadecenoic acid | 7.404 | 1 | 6.509‧10^-3^ | 1.656 |
| Linoleic acid | 11.586 | 1 | 6.644‧10^-4^ | 1.595 | Tyrosine | 7.115 | 1 | 7.645‧10^-3^ | 1.634 |
| Tryptophan | 10.893 | 1 | 9.652‧10^-4^ | 1.569 | Pyroglutamic acid | 7.056 | 1 | 7.898‧10^-3^ | 1.629 |
| 11Z-Eicosenoic acid | 10.078 | 1 | 1.501‧10^-3^ | 1.281 | D-2-Aminobutyric acid | 6.448 | 1 | 1.111‧10^-2^ | 1.578 |
| Vaccenic acid | 8.530 | 1 | 3.493‧10^-3^ | 1.453 | Glutamic acid | 5.483 | 1 | 1.920‧10^-2^ | 1.488 |
| Arachidonic acid | 8.239 | 1 | 4.099‧10^-3^ | 1.262 | Ornithine | 5.033 | 1 | 2.487‧10^-2^ | 1.256 |
| Palmitoleic acid | 7.309 | 1 | 6.860‧10^-3^ | 1.375 | Methionine | 4.688 | 1 | 3.037‧10^-2^ | 1.402 |
| gamma Linolenic acid | 7.210 | 1 | 7.248‧10^-3^ | 1.262 | Lysine | 4.545 | 1 | 3.301‧10^-2^ | 1.351 |
| Adrenic acid | 6.424 | 1 | 1.126‧10^-2^ | 1.011 | Cysteine | 4.054 | 1 | 4.407‧10^-2^ | 1.324 |
| alpha Linolenic acid | 5.928 | 1 | 1.490‧10^-2^ | 1.277 |  |  |  |  |  |
| Glutaric acid | 4.959 | 1 | 2.596‧10^-2^ | 1.210 |  |  |  |  |  |
| Tyrosine | 4.705 | 1 | 3.008‧10^-2^ | 1.198 |  |  |  |  |  |
| *cis*-4-Hydroxyproline | 4.203 | 1 | 4.036‧10^-2^ | 1.149 |  |  |  |  |  |
| Malic acid | 4.123 | 1 | 4.230‧10^-2^ | 1.054 |  |  |  |  |  |
| 11,14-Eicosadienoic acid | 4.099 | 1 | 4.291‧10^-2^ | 1.081 |  |  |  |  |  |
| Glycine | 3.842 | 1 | 4.998‧10^-2^ | 1.110 |  |  |  |  |  |

**Table S4** Metabolic pathways identified by pathway analysis with metabolomic data obtained from (a) gill and (b) adductor muscle samples of control and post-harvest simulated mussels (Total cmpd: total number of compounds found in this study involved in each metabolic pathway; Hits: number of differentially expressed metabolites detected in the identified metabolic pathway; FDR: False discovery rate). Ratio represents enrichment ratio obtained from quantitative enrichment analysis.

| Metabolic pathway | Total Cmpd | Hits | *P* value | -log(*p*) | FDR | Impact | Ratio |
| --- | --- | --- | --- | --- | --- | --- | --- |
| (a) Gill |  |  |  |  |  |  |  |
| Citrate cycle (TCA cycle) | 6 | 3 | 2.005‧10^-5^ | 4.698 | 3.102‧10^-4^ | 0.077 | 6.778 |
| Pyruvate metabolism | 3 | 2 | 4.007‧10^-5^ | 4.397 | 3.102‧10^-4^ | 0.040 | 5.577 |
| Alanine, aspartate and glutamate metabolism | 6 | 3 | 7.700‧10^-5^ | 4.114 | 3.102‧10^-4^ | 0.196 | 7.564 |
| Propanoate metabolism | 2 | 1 | 9.125‧10^-5^ | 4.040 | 3.102‧10^-4^ | 0.000 | 9.181 |
| Butanoate metabolism | 1 | 1 | 9.125‧10^-5^ | 4.040 | 3.102‧10^-4^ | 0.000 | 9.181 |
| Glycolysis / Gluconeogenesis | 1 | 1 | 2.273‧10^-4^ | 3.644 | 6.439‧10^-4^ | 0.101 | 8.685 |
| Tryptophan metabolism | 1 | 1 | 1.133‧10^-2^ | 1.946 | 2.751‧10^-2^ | 0.235 | 5.471 |
| alpha-Linolenic acid metabolism | 1 | 1 | 5.183‧10^-2^ | 1.285 | 9.881‧10^-2^ | 1.000 | 3.621 |
| Arachidonic acid metabolism | 1 | 1 | 5.506‧10^-2^ | 1.259 | 9.881‧10^-2^ | 0.000 | 3.541 |
| Tyrosine metabolism | 2 | 1 | 7.139‧10^-2^ | 1.146 | 9.881‧10^-2^ | 0.391 | 4.608 |
| Phenylalanine, tyrosine and tryptophan biosynthesis | 2 | 1 | 7.139‧10^-2^ | 1.146 | 9.881‧10^-2^ | 0.000 | 3.189 |
| Glyoxylate and dicarboxylate metabolism | 7 | 2 | 8.578‧10^-2^ | 1.067 | 9.881‧10^-2^ | 0.077 | 2.603 |
| Arginine and proline metabolism | 5 | 1 | 8.599‧10^-2^ | 1.066 | 9.881‧10^-2^ | 0.131 | 2.932 |
| Glycine, serine and threonine metabolism | 5 | 1 | 9.881‧10^-2^ | 1.005 | 9.881‧10^-2^ | 0.317 | 2.738 |
| Glutathione metabolism | 5 | 1 | 9.881‧10^-2^ | 1.005 | 9.881‧10^-2^ | 0.089 | 2.738 |
| Lipoic acid metabolism | 1 | 1 | 9.881‧10^-2^ | 1.005 | 9.881‧10^-2^ | 0.002 | 2.738 |
| Porphyrin metabolism | 2 | 1 | 9.881‧10^-2^ | 1.005 | 9.881‧10^-2^ | 0.000 | 2.738 |
| (b) Adductor muscle | |  |  |  |  |  |  |
| Alanine, aspartate and glutamate metabolism | 6 | 3 | 7.980‧10^-5^ | 4.098 | 7.940‧10^-4^ | 0.196 | 8.970 |
| Citrate cycle (TCA cycle) | 6 | 1 | 2.269‧10^-4^ | 3.644 | 7.940‧10^-4^ | 0.033 | 9.500 |
| Propanoate metabolism | 2 | 1 | 2.269‧10^-4^ | 3.644 | 7.940‧10^-4^ | 0.000 | 9.500 |
| Butanoate metabolism | 1 | 1 | 2.269‧10^-4^ | 3.644 | 7.940‧10^-4^ | 0.000 | 9.500 |
| Glutathione metabolism | 5 | 2 | 1.656‧10^-2^ | 1.781 | 4.638‧10^-2^ | 0.020 | 3.451 |
| Tyrosine metabolism | 2 | 1 | 2.571‧10^-2^ | 1.590 | 5.142‧10^-2^ | 0.391 | 5.326 |
| Phenylalanine, tyrosine and tryptophan biosynthesis | 2 | 1 | 2.571‧10^-2^ | 1.590 | 5.142‧10^-2^ | 0.000 | 4.617 |
| Cysteine and methionine metabolism | 5 | 2 | 3.337‧10^-2^ | 1.477 | 5.840‧10^-2^ | 0.323 | 3.216 |
| Glycine, serine and threonine metabolism | 5 | 1 | 8.059‧10^-2^ | 1.094 | 9.402‧10^-2^ | 0.000 | 3.032 |
| Taurine and hypotaurine metabolism | 1 | 1 | 8.059‧10^-2^ | 1.094 | 9.402‧10^-2^ | 0.000 | 3.032 |
| Thiamine metabolism | 1 | 1 | 8.059‧10^-2^ | 1.094 | 9.402‧10^-2^ | 0.000 | 3.032 |
| Pantothenate and CoA biosynthesis | 2 | 1 | 8.059‧10^-2^ | 1.094 | 9.402‧10^-2^ | 0.000 | 5.645 |
| Arginine biosynthesis | 3 | 1 | 9.944‧10^-2^ | 1.002 | 9.944‧10^-2^ | 0.250 | 5.494 |
| Arginine and proline metabolism | 5 | 1 | 9.944‧10^-2^ | 1.002 | 9.944‧10^-2^ | 0.148 | 2.729 |

**Table S5** Significantly different metabolites from (a) gills and (b) adductor mussels of mussels after different treatments.

| (a) Gill |  |  |  | (b) Adductor muscle |  |  |  |
| --- | --- | --- | --- | --- | --- | --- | --- |
| Metabolite | *χ*^2^ | *df* | *p* value | Metabolite | *χ*^2^ | *df* | *p* value |
| Succinic acid | 72.496 | 4 | 6.743‧10^-15^ | Succinic acid | 79.632 | 4 | 2.085‧10^-16^ |
| Phosphoenolpyruvic acid | 38.081 | 4 | 1.078‧10^-7^ | Fumaric acid | 59.050 | 4 | 4.593‧10^-12^ |
| Glutathione | 35.824 | 4 | 3.145‧10^-7^ | Arachidonic acid | 45.299 | 4 | 3.446‧10^-9^ |
| Alanine | 33.194 | 4 | 1.090‧10^-6^ | Malic acid | 43.200 | 4 | 9.403‧10^-9^ |
| Serine | 28.863 | 4 | 8.335‧10^-6^ | Alanine | 43.053 | 4 | 1.009‧10^-8^ |
| Histidine | 28.368 | 4 | 1.050‧10^-5^ | Phosphoenolpyruvic acid | 30.577 | 4 | 3.734‧10^-6^ |
| Strombine | 33.913 | 4 | 7.763‧10^-7^ | Tyrosine | 20.846 | 4 | 3.398‧10^-4^ |
| Glutamic acid | 25.696 | 4 | 3.645‧10^-5^ | D-2-Aminobutyric acid | 19.401 | 4 | 6.555‧10^-4^ |
| Ornithine | 21.049 | 4 | 3.097‧10^-4^ | Aspartic acid | 19.166 | 4 | 7.291‧10^-4^ |
| 4-Hydroxyphenylacetic acid | 20.596 | 4 | 3.808‧10^-4^ | Glutamic acid | 15.181 | 4 | 4.341‧10^-3^ |
| Tryptophan | 20.189 | 4 | 4.582‧10^-4^ | Ornithine | 14.938 | 4 | 4.831‧10^-3^ |
| Arachidonic acid | 19.979 | 4 | 5.043‧10^-4^ | Pyroglutamic acid | 14.402 | 4 | 6.117‧10^-3^ |
| Linoleic acid | 18.060 | 4 | 1.201‧10^-4^ | Lysine | 13.178 | 4 | 1.044‧10^-2^ |
| 11,14-Eicosadienoic acid | 17.307 | 4 | 1.685‧10^-4^ | Strombine | 12.328 | 4 | 1.507‧10^-2^ |
| Docosadienoate (22_2n6) | 17.236 | 4 | 1.739‧10^-4^ | Cysteine | 10.389 | 4 | 3.437‧10^-2^ |
| Asparagine | 16.662 | 4 | 2.248‧10^-3^ | 4-Hydroxyphenylacetic acid | 11.952 | 4 | 1.771‧10^-2^ |
| Vaccenic acid | 16.291 | 4 | 2.652‧10^-4^ | Capric acid | 11.067 | 4 | 2.582‧10^-2^ |
| Docosahexaenoic acid | 16.280 | 4 | 2.666‧10^-3^ | Glutamine | 10.981 | 4 | 2.678‧10^-2^ |
| gamma Linolenic acid | 15.895 | 4 | 3.163‧10^-3^ | Asparagine | 10.751 | 4 | 2.951‧10^-2^ |
| 11Z-Eicosenoic acid | 15.860 | 4 | 3.212‧10^-3^ | Malonic acid | 10.067 | 4 | 3.931‧10^-2^ |
| Proline | 15.684 | 4 | 3.474‧10^-3^ | Methionine | 9.523 | 4 | 4.929‧10^-2^ |
| Fumaric acid | 14.598 | 4 | 5.612‧10^-3^ |  |  |  |  |
| Docosapentaenoic acid (22n_3) | 13.947 | 4 | 7.465‧10^-3^ |  |  |  |  |
| Eicosapentaenoic acid | 13.939 | 4 | 7.493‧10^-3^ |  |  |  |  |
| D-2-Aminobutyric acid | 10.719 | 4 | 2.991‧10^-2^ |  |  |  |  |
| Adrenic acid | 12.835 | 4 | 1.211‧10^-2^ |  |  |  |  |
| Pyroglutamic acid | 12.764 | 4 | 1.249‧10^-2^ |  |  |  |  |
| Aspartic acid | 12.084 | 4 | 1.673‧10^-2^ |  |  |  |  |
| Pentadecanoic acid | 10.817 | 4 | 2.870‧10^-2^ |  |  |  |  |
| Palmitoleic acid | 10.629 | 4 | 3.110‧10^-2^ |  |  |  |  |
| Valine | 9.770 | 4 | 4.449‧10^-2^ |  |  |  |  |
| Dodecanoic acid | 9.618 | 4 | 4.737‧10^-2^ |  |  |  |  |
| Malonic acid | 9.521 | 4 | 4.931‧10^-2^ |  |  |  |  |
